# Thoughts on Transfusion

**Published:** 1854-05

**Authors:** 


### ART. V.—*Thoughts on Transfusion.*

By Rusticus.

You told me, O Editor! that in a post-mortem examination of a certain case of Purpura Hæmorrhagica, you found the body so drained of blood that its tissues were blanched as if by some decolorizing injection — that the brain was hard, and white, and dry, that the veins of the pia mater were empty, the choroid plexus a white and colorless net work, that no red points were seen on slicing the cerebral matter, and, finally, that the sinuses, those firm, unyielding receptacles, were themselves as empty as an artery. Without stopping to dwell upon the refutation which this case offers of that theory which makes the cranial cavity an unyielding case, incapable of any variation in the quantity of blood within it — albeit it is more conclusive than any of Burrows experiments on the cerebral circulation — I would, after pausing for a moment to notice one or too other facts in the autopsy, go on to reason somewhat about the treatment which might have been made available in this fatal case.

Of course the spots of purpura which covered the body do not differ from other cases. The lungs, you say, were healthy, except an unusual hypostatic congestion; that the heart presented no unusual appearance that the stomach, though containing some disorganized blood, had only some punctated red spots to indicate disease, and that its mucous membrane was as firm as usual. In the mesentery you found a singular enlargement of the glands, with occasional interstitial hæmorrhages. The uterus contained blood, which, like the blood in every other part of the system, was disorganized.

But in all this list of organs I find no record of organic disease, save in the mesenteric glands. What conclusions may we draw from all these interesting facts?

Certainly, good Editor! there was one very sound and sensible conclusion in the remark you say was made by the attending physician, viz., that "it was a good case for the transfusion of blood."

I agree with him. But first let us look over what little is known of the pathology of purpura hæmorrhagica, with two points in view. I want to know first; where is the disease? in the tissues or the fluids? and second; what is its cause? This latter knowledge I only hope to attain proximately.

The autopsy you mention (with many others on record) proves this: that these hæmorrhages may occur in any tissue; in the muscles, in the skin, in the serous or mucous membranes, or in the fibrous tissues. Is it, then, probable that one disease shall thus affect all tissues? Can we call it a disease of the capillary system, when capillaries differ so much in shape, length, direction, and construction, as they do in all these various tissues? Can any *organic* disease, (save those accompanied by destructive ulceration,) thus attack all organs? Again, does not your post-mortem show a healthy condition of the tissues themselves? It seems to me that this case, like others, shows that the capillaries would have had no difficulty in transmitting *good* blood through to the veins, and that the depraved condition of the blood is the only cause of hæmorrhage. Look at the blood itself. Drawn off by venesection, it does not separate into a clot swimming in serum, but is a homogeneous, friable, blackish mass of imperfectly organized clot, destitute of serum.

Look again at those cases which recover, or, to make it stronger, at those local recoveries which take place in fatal cases. We have an ecchymosis beneath the skin, not differing from a bruise in its pathology; and we see it absorbed; the skin growing first green, then yellow, till it assumes its natural color.

Why? The part itself is healthy. The effused blood is a foreign body capable of absorption. If the part itself was enfeebled, we should have no absorption.

I shall therefore issue my *ipse dixit*. Purpura is a blood disease. All sensible men understand this, so that I do not lay claim to a discovery. Yet in many authors you find purpura classed among cutaneous diseases.

I know of only two ways in which blood diseases may originate. The first is in defective nutrition or assimilation; the second by the zymotic action of foreign substances introduced by contact, inhalation, or otherwise.

We can hardly suppose the latter to have any anything to do with purpura. If so, it would occur more frequently, and would show a disposition to run through families.

But there is a difficulty in supposing a defect of nutrition as the cause. Scurvy depends upon deficiency of the tartaric, malic, or citric acids; but we have no reason to suppose any deficiency of these in a potato-eating Irishwoman, like Dr. R.'s patient. That vegetable has a large amount of one or the other of these acids, existing in combination with potash.

I do not find mesenteric disease mentioned as a common occurrence in purpura. But I am half inclined to consider it as one of the causes, and to ascribe to deranged assimilation a principal share in the causation of purpura. What we want is a chemical analysis of the blood and secretions in this disease.

This much we do know. The system may become so enfeebled as to be unable to manufacture good blood, and we know further, that could we secure the presence in the circulation of a moderate quantity of good blood, it would be the most efficient tonic we could exhibit; supplying an exhausting waste, and sending new life to the wearied heart, deprived of its healthy and accustomed stimulus.

I will not stop to discuss the treatment of purpura. I have seen cases get well nicely on hæmostatics, such as the gallic acid with port wine. The books tell us that we may bleed, (*English* books mind you! blood-thirsty, sanguinary emanations from old Fogy-dom,) and calomel has been lauded in connection with brisk purgations, though why, Lord only knows!

But I wish to talk of the transfusion of blood, as suggested by your friend.

We know in these days but little of it. Now and then we hear of some women snatched from the very sill of the door of death from flooding, by the inpouring of another's vitality into her veins. Then years pass, and we hear no more.

The best literature on this subject is a hundred and fifty years old, the best experiments were made by Sir Christopher Wren, before he abandoned physic for architecture, or built for his monument the mighty dome of St. Paul's Church. Let us look up the old and musty records. I suppose it must have been in the resurrection exploits of student life that I acquired such a taste for church-yard literature, such a proclivity to stir up the dry bones of dead and gone antiquities.

Before the time of Sir Christopher Wren, and as early as 1615, Libavius describes the operation of transfusion very plainly; but it would seem that to

Sir Christopher is due the credit of the invention of injecting the veins with different fluids, while it seems that Lower (1660) performed this operation of transfusion very skillfully.

One of his experiments consisted in arranging two dogs so that the blood should flow through a tube from the carotid of one into the jugular vein of the other, an exit being provided by an opening in the opposite jugular. Thus the dog whose carotid was opened was bled to death through the other ; the latter being able to run off, after shaking himself, apparently uninjured.

As an instance we quote from Lower :

"I once *bled* a Mastiff into a Cur, and the little Dog bled out at least double the Quantity of his own *Blood*, and left the Mastiff dead upon the Table. And after he was untied, he ran away and shaked himself, as if he had only been thrown into water."

The connecting medium between the two circulations, in Lower's experiments, was a goosequill.

Sir Edmund King took 49 oz. of blood from a sheep, and when it seemed likely to die, he connected the bleeding vein with the jugular vein of a calf, and restored it to full strength immediately.

Mr. Thomas Coxe bled a mangy dog into a healthy one. The mangy dog was cured, while the healthy one remained as well as ever. Ergo mange is not a blood disease.

"*Mr. Gayant transfused the Blood of a Young* Dog into the *Veins* of an *Old*, which two Hours after did leap and frisk ; whereas he was almost blind with age, and could hardly stir before."

Mr. Denys (the inventor of the styptic) holds forth thus.

"Since the 9th of *March* $166\frac{6}{7}$, we have *transfused* the *Blood* of three Calves into three Dogs. After which, the Dogs (all of them) did eat as well as before : And one of the three Dogs, from which so much *Blood* had been drawn the Day before that he could hardly stir any more, having been supply'd the next Morning with Blood of a Calf, recover'd instantly his Strength, and shew'd a surprizing Vigour."

In Italy, at Bononia, May 8th, 1677, S. Cassini, and at Udine, May 20th, 1668, S. Griffoni performed similar experiments with equal success. Sir Edmund King, about this time, suggested the injection of the blood of beasts into that of men. He describes a silver tube adapted to the purpose. I give at some length his description of a transfusion performed by himself and Richard Lower. It is worthy of notice that all of these experiments seem to take no precautions against the entrance of air.

"The Experiment of *transfusing Blood* in a *humane Vein*, was perform'd upon Mr. *Arthur Coga*, Nov. 23, 1667, after this manner: Having prepared the *Caroted Artery* in a young Sheep, we made a Incision in the *Vein*,* observing the Method above-mention'd, without any Alteration, but in the Shape of one of our Pipes, which we found more convenient for our purpose. And having opened the *Vein* in the *Man's* Arm, with as much Ease as in the common way of Venæ-section, we let thence run out six or seven *Ounces* of *Blood*. Then we planted our Silver Pipe into the said Incision, and inserted Quills between the two Pipes already advanced in the two Subjects, to convey the *Arterial Blood* from the Sheep into the *Vein* of the *Man*. The *Blood* ran freely into the *Man's vein*, for the Space of two Minutes at least; so that we could feel a *Pulse* in the said *Vein* just beyond the Silver Pipe. * * * * * We judg'd that about 9 or 10 Ounces were receiv'd into the *Man's Veins*. The *Man* after the *Operation*, as well as in it, found himself *very well*."

The most daring of these experiments were made by Fabritius at Dantzic. Here purgative medicies were injected into the veins of patients. One, a venereal patient, had ʒij of some laxative thrown into the veins. He was said to be cured. Two others, epileptics, had "injected into their *Veins Laxative Rosin* dissolved in an *Anti-Epileptical* Spirit." One was cured; the other "by going into the Air, and taking Cold, and not observing any Diet, cast herself away." In all these cases there was a cathartic movement.

I could bring down this history to a later date, and show that so far as any information exists, the transfusion of blood has ever been a safe operation, even in the hands of those unaware of its greatest danger, viz. the introduction of air into the vein.

What need of a sermon? You Mr. Editor must see, and so must your readers, that the transfusion of blood might safely be a much more frequent operation than it now is. I do not think that a complicated apparatus would be needed, or desirable. Any use of long tubes would increase the danger of admitting air, and it seems to me that a glass syringe inverted before injecting, so as to exclude all air from its chamber, and inserted into a single opening into the cephalic vein, guarded by the fingers of an assistant, is all the instrument necessary.

The apparent heroic character of the operation is the principal barrier to its use, but as it is only called for in desperate cases, and really requires no

---

*Evidently, artery is here intended as the distinction was then known between arteries and veins.

great skill or tact, I wonder that it has so fallen into disuse. It is, mine Editor, one of those direct and straightforward *cures*, such as gladden the heart of a true physician, and give him faith in the resources of his art.

Who can imagine a greater triumph than that afforded in such a case as we may suppose. Here is a woman whose life-blood has ebbed from her till the faint sound of the heart is scarcely heard, cold, pale, and pulseless, she lies in a syncope so like to death, that soon it will merge into the eternal faintness. Here, whence hope has fled, the heroic remedy would come as does the life-boat to the drowning man.

——— × Roads.

